# AAV gene therapy vectors in the TMJ

**DOI:** 10.1002/cre2.636

**Published:** 2022-07-24

**Authors:** Sabine M. Brouxhon, Michael Kerry O'Banion, Stephanos Kyrkanides

**Affiliations:** ^1^ Department of Physiology, School of Medicine Stony Brook University Stony Brook New York USA; ^2^ Departments of Neuroscience and of Neurology, School of Medicine & Dentistry University of Rochester Medical Center Rochester New York USA; ^3^ Department of Oral Health Science, College of Dentistry University of Kentucky Lexington Kentucky USA

**Keywords:** gene therapy, TMJ, trigeminal nerve, vectors

## Abstract

**Objectives:**

The goal of this project was to evaluate the use of two adeno‐associated viral vector serotypes, adeno‐associated viral vectors (AAV)‐2 and AAV‐6, approved for and used for gene therapy in humans, for the delivery of therapeutic genes to the temporomandibular joint (TMJ) and the attendant sensory nerves.

**Methods:**

Young adult wild‐type C57BL/6 mice were intra‐articularly inoculated with AAV‐2 and AAV‐6 encoding the reporter gene *gfp*, the expression of which was assessed in the TMJ as well as along nerves innervating the TMJ.

**Results:**

AAV‐2 and AAV‐6 serotypes were characterized by varying levels of tissue tropism demonstrating different efficacy of infection for articular chondrocytes, meniscal fibroblasts, and trigeminal neurons. Specifically, AAV‐2 infected both neurons and articular chondrocytes/meniscal fibroblasts, whereas AAV‐6 showed selectivity primarily for neurons.

**Conclusions:**

The results of this study are clinically significant in the successful application of gene therapy vectors for TMJ disorders, as this new knowledge will allow for appropriate targeting of specific therapeutic genes to selective tissues (neurons vs. chondrocytes/fibroblasts) as needed by using specific viral vector serotypes.

## 1 INTRODUCTION

Gene therapy is a treatment that involves the administration of specific nucleic acids to modify gene expression in patients. The concept of gene therapy is almost 40 years old, and the first meaningful studies were done around 30 years ago (Rosenberg et al., 1990). Recent advances in transfer vectors have led to the approval of two gene therapies by the Food and Drug Administration (FDA), including Luxturna in 2017 for inherited retinal dystrophy and Zolgensma in 2019 for spinal muscular atrophy. Moreover, there are approximately 900 investigational new drug (IND) applications for gene therapy clinical studies. Many of these gene therapies have been developed based on adeno‐associated viral vectors (AAV).

After more than 25 years of development, gene therapy for arthritis has finally entered clinical practice. In South Korea, gene therapy was approved for the treatment of osteoarthritis, and other gene therapeutics are in the pipeline elsewhere (Deviatkin et al., 2020; Evans et al., 2018). Gene therapy has been tested in joint dysfunction and arthritis animal models. Opioid precursor genes, such as enkephalins and β‐endorphin or its receptors, have been successfully transferred to afferent sensory neurons via intrathecal administration, as well as direct injections into dorsal root ganglia or the sciatic nerve (Beutler et al., 1995; Braz et al., 2001; Finegold et al., 1999; Goss et al., 2001; Guedon et al., 2015; Ogawa et al., 2018; Pohl & Braz, 2001; Towne et al., 2009; Xu, Gu, Li, et al., 2003, Xu, Gu, Xu, et al., 2003). These studies resulted in enhanced production of opioid peptides or receptors, and a reduction of hyperalgesia in various nociceptive models. However, despite efficacious outcomes, these previous studies depended on direct injections of viral vectors into sensory ganglia or the spinal cord, and neurosurgical procedures that are invasive in nature and associated with possible morbidity. Moreover, intrathecal, intra‐ganglionic, or nerve fiber injections do not address the location of pain origin, but rather, randomly cover an expansive area of sensory input.

With the emergence of gene therapy for joint disorders, it is plausible that in the near future similar treatments will become available for temporomandibular joint disorders. In a mouse model of temporomandibular joint (TMJ) inflammation (Lai et al., 2006), we previously developed and tested an experimental feline immunodeficiency viral (FIV) vector for the transfer of the human μ‐opioid receptor (Kyrkanides et al., 2007). However, FIV vectors have yet to be approved by the FDA for use in humans. Li et al. (2009) reported effective infection of the TMJ disc, glenoid fossa, and condylar cartilage by AAV2 serotype vector transferring the reporter gene eGFP. However, it is unclear from their work whether AAV2 can successfully infect trigeminal neurons. Based on the work of Xu, Gu, Li, et al. (2003) and Xu, Gu, Xu, et al. (2003), we know that AAV2 is neurotropic when injected directly into ganglia or neuronal bundles, but it is not known whether this vector can infect neurons via their free nerve endings.

The goal of this project was to evaluate the use of two different AAV serotype vectors approved for and already used for gene therapy in humans for the delivery of therapeutic genes to the TMJ, in particular AAV2 and AAV6. To accomplish our goal, we employed different serotypes of the AAV vector, transferring the reporter gene green fluorescent protein (gfp) to the TMJ of mice via intra‐articular administration. Our study closes the scientific gap in this area with the generation of new knowledge that will allow the development of gene therapies for TMJ disorders.

## 2 MATERIALS AND METHODS

### 2.1 Viral vectors

The adeno‐associated viral vectors (AAV) transfer vector for the reporter gene green fluorescent protein (gfp) was constructed by Wang et al. (2003) and packaged for this study by the Core Facility of the National Institute of Drug Abuse in different serotypes (type ‐2 and ‐6,) as previously described (Howard et al., 2008), and kindly donated to S. K.

### 2.2 Vertebrate animals

The protocols, including vertebrate animals, were reviewed and approved by the University of Rochester IACUC (UCAR Protocol # 2003‐191). A total of five male and five female wild‐type C57BL/6 mice were purchased from JAX (Bar Harbor, ME) and housed in the University of Rochester Vivarium facility for 2 weeks for acclimation. The mice (*N* = 5) were randomly assigned to two groups, each to test a different AAV serotype (types ‐2 and ‐6).

#### 2.2.1 Anesthesia

Ketamine (40 mg/kg) administration via intraperitoneal injections was used for anesthesia.

#### 2.2.2 Virus administration

Mice were injected with 10 µl of an aqueous solution containing 10^12^ infectious AAV particles using a fine needle (33 GA) under a surgical plane of anesthesia. The needle was inserted into the TMJ from a posterior‐inferior direction and solutions were injected into the superior joint space. The mice consistently showed no signs of distress or discomfort. The mice were returned to their cages after viral inoculation.

#### 2.2.3 Hazard control

Personnel observed a BSL‐2 level of precaution when injecting viral particles into suitably anesthetized animals, and the skin of the injected animals was treated with alcohol/iodine to inactivate any potential virus on the skin surface before removal from the laminar flow hood. All materials related to the injections were disposed of in biohazard‐approved receptacles and autoclaved in accordance with Biosafety Office's recommendation. All genes and vectors used were registered with the Institutional Biosafety Committee. In addition, our laboratory underwent annual inspections by the institutional chemical hygiene officer and complied with NIH and institutional guidelines.

#### 2.2.4 Euthanasia

The mice were euthanized 5 weeks after intra‐articular TMJ inoculation by CO_2_ inhalation followed by decapitation.

### 2.3 TMJ histopathology

After the mice were euthanized, their heads were harvested, de‐fleshed, and immersed in 10% formalin solution for fixation. Subsequently, the specimens were decalcified in ethylenediaminetetraacetic acid solution, processed, and paraffin‐embedded. Histology TMJ sections were cut and collected onto glass slides, deparaffinized, and subsequently analyzed as previously described (Lai et al., 2006). Serial parasagittal sections collected every 100 µm covering the entire TMJ condyle were evaluated under ×40 magnification. First, the TMJ sections were stained by H&E. Second, GFP protein was detected by immunohistochemistry using a rabbit anti‐gfp polyclonal antibody as previously described (Fiorentino et al., 2008). The sections were then viewed under an Olympus BX51 light microscope for the presence of immune‐positive structures. Images were captured using an attached CCD digital camera.

### 2.4 Brain stem and trigeminal ganglia analyses

After the mice were euthanized, their brain stems and trigeminal ganglia were harvested and fixed by immersion into 10% formalin solution (Fiorentino et al., 2008). In brief, brain stems were sectioned horizontally at 18 µm on a freezing cryostat. Sections every 180 μm collected serially on glass slides covering the entire region of interest (−5 mm to +10 mm relative to obex for the brain stem and the entire trigeminal ganglia) were included from each animal. The sections were then analyzed under an Olympus BX51 fluorescent microscope for the presence of GFP^+^ cell bodies. Images were acquired using an attached CCD digital camera.

## 3 RESULTS

### 3.1 AAV serotype tropism toward TMJ articular tissues

**Figure 1:**
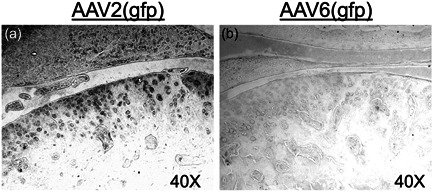
AAV‐2, but not AAV‐6, efficiently infects chondrocytes and fibroblasts in the temporomandibular joint (TMJ). Expression of the reporter gene *gfp* was evaluated by immuno‐histochemistry in TMJ sections. (a) AAV‐2 resulted in expression of the reporter gene *gfp* in articular chondrocytes and meniscal fibroblasts, but not (b) AAV‐6. AAV, adeno‐associated viral transfer vectors.

Immunohistochemical analysis of TMJ histology sections for the reporter gene *gfp* revealed that AAV‐2, but not AAV‐6, serotype is capable of infecting the articular chondrocytes and meniscal fibroblasts (Figure 1).

### 3.2 AAV serotype tropism toward nerve fibers in the TMJ

**Figure 2:**
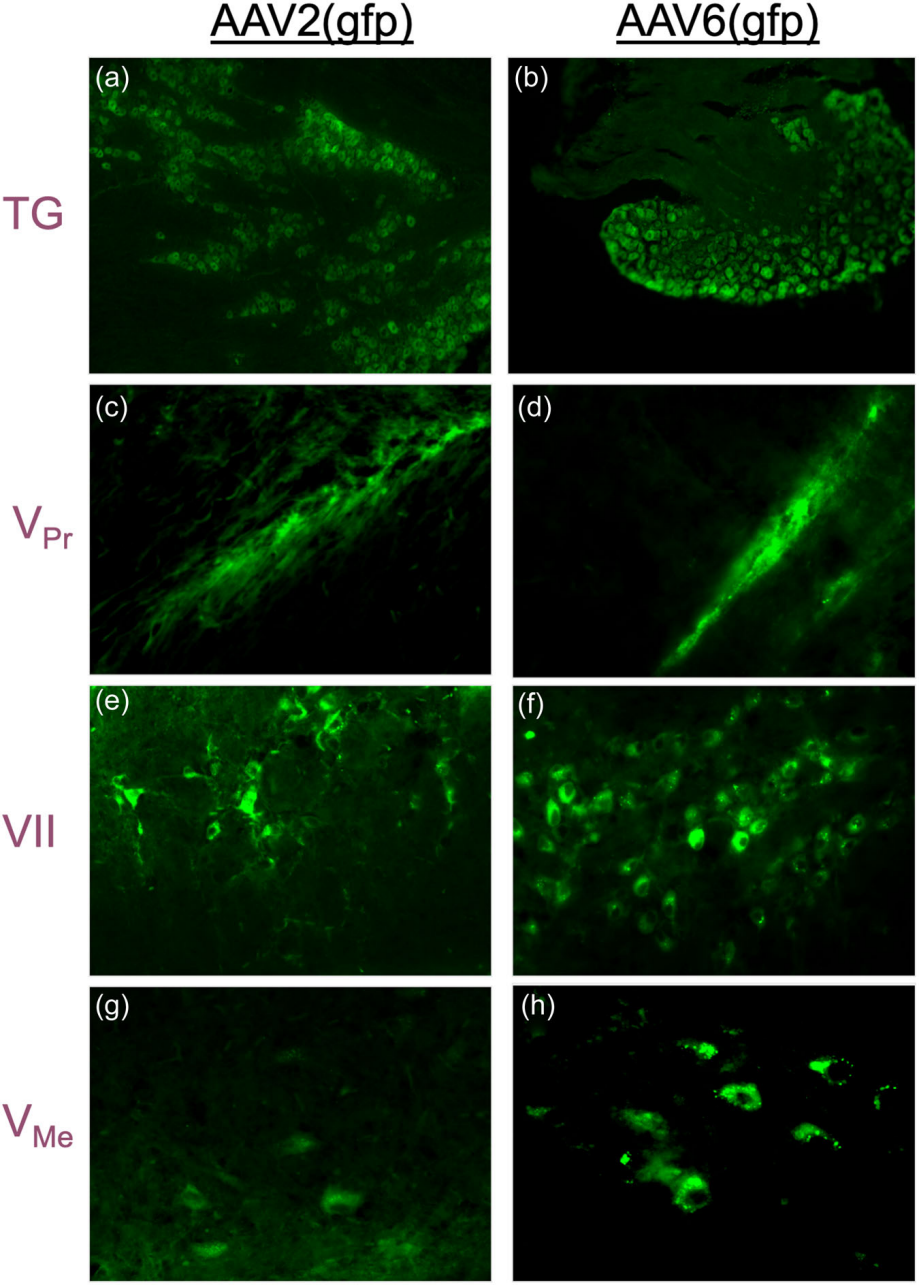
Expression of *gfp* in the central nervous system. Expression of the reporter gene *gfp* was evaluated under fluorescent microscopy in the brain stem of mice that received intra‐articular injection of AAV(gfp) in the temporomandibular joint (TMJ). (a, b) Representative images of trigeminal ganglia harvested from mice that received AAV2 and AAV6, respectively, in the TMJ. (c, d) Principal nucleus of the trigeminal nerve of mice that received AAV2 and AAV6, respectively. (e, f) Facial nerve nucleus of mice that received AAV2 and AAV6, respectively. (g, h) Mesencephalic nucleus of the trigeminal nerve of mice that received AAV2 and AAV6, respectively. AAV, adeno‐associated viral transfer vectors.

Analysis of *gfp* expression under a fluorescent microscope revealed that both AAV‐2 and AAV‐6 serotypes effectively infected trigeminal nerves after intra‐articular administration, presumably through uptake by the free nerve endings, with evidence of retrograde transport of the transfer vector to nuclei in the neuronal cell bodies located in the Gasserion ganglion of the trigeminal nerve (TG) and the facial nerve nucleus (VII) for the facial nerve. Fluorescence of GFP was also detected along the trigeminal axis, including neuronal cell bodies located in the trigeminal ganglia and proximally in the principal trigeminal nucleus (V_Pr_) and the mesencephalic trigeminal nucleus (V_Me_). Remarkably, AAV‐2 and AAV‐6 efficiently infected both trigeminal and facial nerve nerves (Figure 2), as GFP was found to be expressed in the trigeminal ganglion (TG) as well as the nucleus of the facial nerve (VII).

## 4 DISCUSSION

Advances in AAV vector safety and efficacy have enabled the FDA to recently approve two gene therapies, including Luxturna in 2017 for inherited retinal dystrophy and Zolgensma in 2019 for spinal muscular atrophy. Moreover, a gene therapy was approved for the treatment of osteoarthritis and has entered clinical practice in South Korea. Predictably, gene therapy for TMJ disorders is in the foreseeable future. To this end, previous work in our laboratory using FIV vectors (Kyrkanides et al., 2007) demonstrated, in a mouse model of TMJ inflammation (Lai et al., 2006), that infection of TMJ tissues and trigeminal nerve endings after intra‐articular administration significantly alleviated the attendant nocifensive behavior. Interestingly, we also observed amelioration of the attendant hard tissue pathology (Kyrkanides et al., 2007), which resulted in part from gene therapy inhibition of neurogenic inflammation in the TMJ (Fiorentino et al., 2008). However, FIV vectors are not FDA approved for human use, whereas AAV vectors are. The goal of this research project was to assess the effectiveness of AAV vectors of different serotypes in transferring exogenous genes to the TMJ and the free nerve endings therein.

We employed two different AAV serotypes in our experiments, AAV‐2 and AAV‐6. Interestingly, our results demonstrate different levels of tropism for different cell types for these vectors. Specifically, we found that AAV‐2 can effectively infect articular chondrocytes, meniscal fibroblasts, and trigeminal sensory nerves via intra‐articular injection into the TMJ. In contrast, AAV‐6 showed a selective preference for neurons, but not for articular chondrocytes or meniscal fibroblasts.

**Figure 3:**
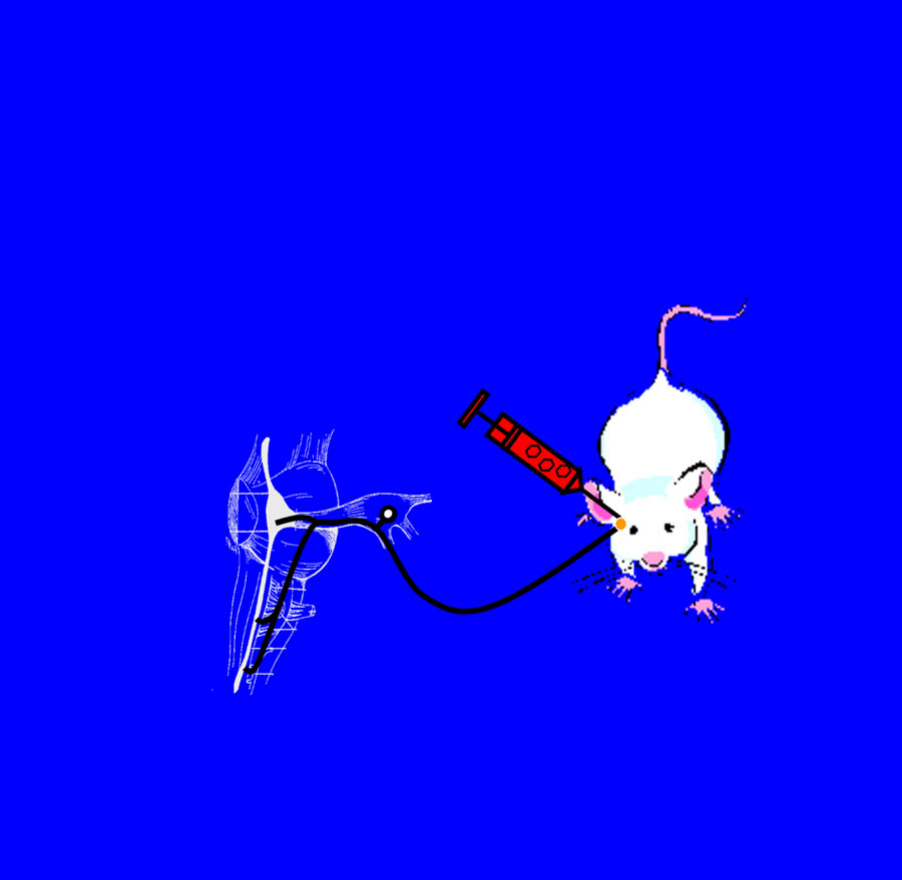
Temporomandibular joint (TMJ) intra‐articular administration of gene therapy vectors. Injection of AAV in the TMJ resulted in infection of free nerve endings of the trigeminal and facial nerves, and retrograde transfer to the nuclei of the neurons located in the trigeminal ganglion and facial nerve nucleus, respectively. There, the reporter gene was expressed and detected in various segments of the trigeminal nuclear complex, including the primary as well as the mesencephalic nucleus of the trigeminal nerve.

Our results further demonstrate that both AAV‐2 and AAV‐6 efficiently transduce trigeminal neurons with the reporter gene *gfp* following intra‐articular inoculation, presumably after infecting free nerve endings (Figure 3). Previous studies reported such neurotropism of AAV‐6 in rat neurons cultured in vitro (Howard et al., 2008). However, we are the first to describe the ability of viral vectors, including FIV (Kyrkanides et al., 2004) in the past and AAV here, to exhibit retrograde uptake by sensory nerve endings in vivo. In contrast, previous attempts required intra‐neuronal or intra‐ganglionic administration, which are both clinically challenging approaches. Conversely, intra‐articular injections into the TMJ are routinely performed by oral surgeons.

Our results are very significant for the successful clinical application of AAV vectors, as they will allow scientists and clinicians to selectively target therapeutic genes to specific tissues (nerves vs. chondrocytes/fibroblasts) as needed. For example, the transfer of a therapeutic transgene to sensory neurons may be beneficial for treating TMJ disorders, but at the same time may be detrimental if transferred to articular chondrocytes and/or meniscal fibroblasts. Taken together, our findings provide important information for making relevant educated decisions when designing gene therapy strategies for the TMJ. In conclusion, AAV vector tissue tropism is an important characteristic, knowledge of which provides a significant advantage in designing gene therapy strategies for TMJ disorders.

As for the next steps, now that the tropism of the AAV‐2 and AAV‐6 vectors has been identified, we will clone the human μ‐opioid therapeutic gene as previously developed (Kyrkanides et al., 2007) into the AAV vectors and evaluate their therapeutic efficacy in alleviating orofacial nociception after intra‐articular administration in the TMJ, and its biodistribution to off‐target sites in the mouse (Lai et al., 2006) as previously described (Kyrkanides et al., 2007). These data will subsequently be employed in preparing an IND application with the FDA, which when approved, will allow us to proceed with Phase‐I clinical trials in human patients.

## AUTHOR CONTRIBUTIONS

Sabine M. Brouxhon, Michael Kerry O'Banion, and Stephanos Kyrkanides participated in the execution of the work, analysis of the data, and composition of the manuscript, whereby Stephanos Kyrkanides served as the lead investigator.

## CONFLICT OF INTEREST

The authors declare no conflict of interest.

## Data Availability

Data will be made available to investigators' requests.

## References

[cre2636-bib-0001] Beutler, A. S. , Banck, M. S. , Bach, F. W. , Gage, F. H. , Porreca, F. , Bilsky, E. J. , & Yaksh, T. L. (1995). Retrovirus‐mediated expression of an artificial beta‐endorphin precursor in primary fibroblasts. Journal of Neurochemistry, 64, 475–481. 7830038 10.1046/j.1471-4159.1995.64020475.x

[cre2636-bib-0002] Braz, J. , Beaufour, C. , Coutaux, A. , Epstein, A. L. , Cesselin, F. , Hamon, M. , & Pohl, M. (2001). Therapeutic efficacy in experimental polyarthritis of viral‐driven enkephalin overproduction in sensory neurons. Journal of Neuroscience, 21, 7881–7888. 11588161 10.1523/JNEUROSCI.21-20-07881.2001 PMC6763863

[cre2636-bib-0003] Deviatkin, A. A. , Vakulenko, Y. A. , Akhmadishina, L. V. , Tarasov, V. V. , Beloukhova, M. I. , & Zamyatnin, A. A., Jr. , Lukashev, A. N. (2020). Emerging concepts and challenges in rheumatoid arthritis gene therapy. Biomedicines, 8, 9. 31936504 10.3390/biomedicines8010009 PMC7168286

[cre2636-bib-0004] Evans, C. H. , Ghivizzani, S. C. , & Robbins, P. D. (2018). Arthritis gene therapy is becoming a reality. Nature Reviews Rheumatology, 14, 381–382. 10.1038/s41584-018-0009-5 29743627

[cre2636-bib-0005] Finegold, A. A. , Mannes, A. J. , & Iadarola, M. J. (1999). A paracrine paradigm for in vivo gene therapy in the central nervous system: Treatment of chronic pain. Human Gene Therapy, 10, 1251–1257. 10340556 10.1089/10430349950018238

[cre2636-bib-0006] Fiorentino, P. M. , Tallents, R. H. , Miller, J. N. , Brouxhon, S. M. , O'Banion, M. K. , Puzas, J. E. , & Kyrkanides, S. (2008). Spinal interleukin‐1beta in a mouse model of arthritis and joint pain. Arthritis and Rheumatism, 58(10), 3100–3109. 18821694 10.1002/art.23866

[cre2636-bib-0007] Goss, J. R. , Mata, M. , Goins, W. F. , Wu, H. H. , Glorioso, J. C. , & Fink, D. J. (2001). Antinociceptive effect of a genomic herpes simplex virus‐based vector expressing human proenkephalin in rat dorsal root ganglion. Gene Therapy, 8, 551–556. 11319622 10.1038/sj.gt.3301430

[cre2636-bib-0008] Guedon, J.‐M. G. , Wu, S. , Zheng, X. , Churchill, C. C. , Glorioso, J. C. , Liu, C.‐H. , Liu, S. , Vulchanova, L. , Bekker, A. , Tao, Y.‐X. , Kinchington, P. R. , Goins, W. F. , Fairbanks, C. A. , & Hao, S. (2015). Current gene therapy using viral vectors for chronic pain. Molecular Pain, 11, 27. 25962909 10.1186/s12990-015-0018-1 PMC4446851

[cre2636-bib-0009] Howard, D. B. , Powers, K. , Wang, Y. , & Harvey, B. K. (2008). Tropism and toxicity of adeno‐associated viral vector serotypes 1,2,5,6,7,8,9 in rat neurons and glia *in vitro* . Virology, 372, 24–34. 18035387 10.1016/j.virol.2007.10.007 PMC2293646

[cre2636-bib-0010] Kyrkanides, S. , Fiorentino, P. M. , Gan, Y. , Lai, Y.‐C. , Shaftel, S. S. , Puzas, J. E. , Piancino, M. G. , O'Banion, M. K. , & Tallents, R. H. (2007). Intra‐articular µ‐opioid receptor induction ameliorates arthritic pain and joint pathology. Arthritis and Rheumatism, 56, 2038–2048. 17530644 10.1002/art.22635

[cre2636-bib-0011] Kyrkanides, S. , Kambylafkas, P. , Miller, J. H. , & Tallents, R. H. (2004). Non‐primate lentiviral vector administration in the TMJ. Journal of Dental Research, 83, 65–70. 14691116 10.1177/154405910408300113

[cre2636-bib-0012] Lai, Y.‐C. , Shaftel, S. S. , Miller, J. N. , Tallents, R. H. , Chang, Y. , Pinkert, C. A. , Olschowka, J. A. , Dickerson, I. M. , Puzas, J. E. , O'Banion, M. K. , & Kyrkanides, S. (2006). Intraarticular induction of interleukin‐1beta expression in the adult mouse, with resultant temporomandibular joint pathologic changes, dysfunction, and pain. Arthritis and Rheumatism, 54, 1184–1197. 16572453 10.1002/art.21771

[cre2636-bib-0013] Li, Q. , Dai, J. , & Rabie, A. B. M. (2009). Recombinant adeno‐associated virus serotype 2 (rAAV2)—An efficient vector for gene delivery in condylar cartilage, glenoid fossa and TMJ disc in an experimental study in vivo. Archives of Oral Biology, 54, 943–950. 19683702 10.1016/j.archoralbio.2009.07.005

[cre2636-bib-0014] Ogawa, N. , Terashimab, T. , Okac, K. , Chanc, L. , & Kojima, H. (2018). Gene therapy for neuropathic pain using dorsal root ganglion‐targeted helper‐dependent adenoviral vectors with GAD67 expression. Pain Reports, 3, e695. 30706038 10.1097/PR9.0000000000000695 PMC6344132

[cre2636-bib-0015] Pohl, M. , & Braz, J. (2001). Gene therapy of pain: Emerging strategies and future directions. European Journal of Pharmacology, 429, 39–48. 11698025 10.1016/s0014-2999(01)01304-8

[cre2636-bib-0016] Rosenberg, S. A. , Aebersold, P. , Cornetta, K. , Kasid, A. , Morgan, R. A. , Moen, R. , Karson, E. M. , Lotze, M. T. , Yang, J. C. , Topalian, S. L. , & Topalian, S. L. (1990). Gene transfer into humans—Immunotherapy of patients with advanced melanoma, using tumor‐infiltrating lymphocytes modified by retroviral gene transduction. New England Journal of Medicine, 323, 570–578. 2381442 10.1056/NEJM199008303230904

[cre2636-bib-0017] Towne, C. , Pertin, M. , Beggah, A. T. , Aebischer, P. , & Decosterd, I. (2009). Recombinant adeno‐associated virus serotype 6 (rAAV2/6)‐mediated gene transfer to nociceptive neurons through different routes of delivery. Molecular Pain, 5, 52. 19737386 10.1186/1744-8069-5-52 PMC2747840

[cre2636-bib-0018] Wang, Z. , Ma, H. I. , Li, J. , Sun, L. , Zhang, J. , & Xiao, X. (2003). Rapid and highly efficient transduction by double stranded adeno‐associated virus vectors *in vitro* and *in vivo* . Gene Therapy, 10, 2105–2111. 14625564 10.1038/sj.gt.3302133

[cre2636-bib-0019] Xu, Y. , Gu, Y. , Li, G.‐W. , & Mae, L.‐L. M. , (2003). Efficiencies of transgene expression in nociceptive neurons through different routes of delivery of adeno‐associated viral vectors. Human Gene Therapy, 14, 897–906. 12828860 10.1089/104303403765701187

[cre2636-bib-0020] Xu, Y. , Gu, Y. , Xu, G.‐Y. , Wu, P. , Li, G.‐W. , & Huang, L.‐Y. M. (2003). Adeno‐associated viral transfer of opioid receptor gene to primary sensory neurons: A strategy to increase opioid antinociception. Proceedings of the National Academy of Sciences of the United States of America, 100, 6204–6209. 12719538 10.1073/pnas.0930324100 PMC156350

